# Parasitic infections in Swiss children: Are we overtesting?

**DOI:** 10.1186/s12876-021-01619-6

**Published:** 2021-01-26

**Authors:** Corinne Légeret, Céline Rüttimann, Hans Fankhauser, Henrik Köhler

**Affiliations:** 1 Children’s Hospital of Aarau, Tellstrasse 9, 5001 Aarau, Switzerland; 2 Microbiology, Hospital of Aarau, Tellstrasse 25, 5001 Aarau, Switzerland

**Keywords:** Children, Abdominal pain, Diagnostics, Parasites, Functional pain

## Abstract

**Background:**

A wide variation of causes can lead to gastrointestinal symptoms in children- an infection with parasites is one of them. The expansion of international travel might lead to an increase in testing children for a correspondent infection. Currently there are no guidelines available, which patients should be tested for a possible parasitical infection.

The aim of the study was to characterize Swiss children suffering from intestinal parasites, in order to provide more knowledge for the clinician who should be tested.

**Methods:**

This is a retrospective study of Swiss pediatric patients, whose stools have been tested for parasites and helminths.

**Results:**

A total of 1855 stool samples, belonging to 572 different children with an average age of 7.9 years, were tested within a 10-year period. The prevalence of a positive result was 4.2%, of which all were positive for *Blastocystis*, and 12.5% had a co-infection with *Endolimax nana*.

**Conclusion:**

Immigrants, immune compromised children with diarrhea and pediatric patients with bloody or protracted diarrhea should have 2 different stool specimens examined for a possible parasitical infection.

## Background

Gastrointestinal symptoms such as abdominal pain, diarrhea, constipation, failure to gain weight and vomiting are among of the most common reasons for primary care, emergency department visits or referrals to gastroenterologists [1]. Since causes can range from self-limited minor diseases to more severe or even life-threatening conditions, it can be challenging for the clinician to decide which diagnostic resources to apply. Within this framework many children are tested for a possible protozoan or helminthic infection.

According to the WHO, a quarter of the world’s population is infected with intestinal-(or soil-) transmitted helminths [2], the main species being roundworms (*Ascaris lumbricoides*), whipworms (*Trichuris trichiura*) and hookworms (*Necator americanus* and *Ancylostoma duodenale*). They are transmitted by eggs, which are passed in the feces of infected people, consequently most affected people live in areas that lack adequate sanitation, hygiene and water with the greatest numbers occurring in sub-Saharan Africa, China and East Asia [2].

Pediatric patients acquire the primary infection as they begin interacting with the environment during their preschool years and reach a maximum worm burden for round- and whipworm (transmission via oral ingestion of embryonated eggs) by school age. In contrast, the maximum hookworm and schistosomiasis intensity (infection results from direct percutaneous invasion of larvae) is typically seen in adolescence or young adulthood [3, 4]. Enteric protozoa- mainly *Giardia intestinalis* including *G. lamblia* and *Entamoeba spp*. are frequently isolated in children from developing countries [5], whilst *Blastocystis* and *Dientamoeba fragilis* are mostly detected in children from developed countries mainly [6].

A wide variation of causes can lead to gastrointestinal symptoms in children, an infection with parasites is one of them. Helminth and protozoan infections were thought to affect mainly immigrant communities, refugees and adoptees from endemic regions. However, with the expansion of international travel, global networking and a high prevalence of children living in poverty within wealthy countries, pediatric clinicians in central Europe may be confronted by an increasing amount of parasitic infections.

The aim of the current study was to address the gap in the literature by characterizing pediatric patients in Switzerland, who have been tested for parasitic/helminthic infections, in order to provide more knowledge for European clinicians whom, when and how to test children.

## Methods

This is a retrospective study of pediatric patients between 0 and 18 years, who are under the care of the Children’s hospital Aarau and whose stool has been tested in the last 10 years (December 2008–December 2018) for parasites and helminths. No exclusions. Following informations were extracted from the medical record of each patient included in the study: sex, date of birth, date, amount and result of stool samples tested, the main and a maximum of two further symptoms why the stool was tested, duration of symptoms (more or less than 4 weeks), possible known underlying diagnoses, allergies and regular intake of medication, travels abroad (including destination) in the last 6 months, migration background and further stool tests. Treatment (product and duration) of patients with a positive stool result was also noted.

All fecal specimens were sampled in a tube containing Sodium Acetate Formalin (SAF) and sent to the microbiology laboratory in the hospital of Aarau. Parasite eggs, larvae, and protozoa are then concentrated using the "Para-Pak SpinCon Stool Concentration System". In a first step the fecal specimen is treated with an surfactant and passed through a preliminary screen by gravity flow. The specimen is then forced by centrifugation through a series of two screens with successively smaller mesh. The resulting pellet is then examined for the presence of parasites by standard wet mount procedures. This method remained the same for the entire 10 years.

Chronic abdominal pain was defined as abdominal pain that persisted for more than 4 weeks in the absence of red flags (blood in the stool, nocturnal diarrhea, jaundice, persistent vomiting, hematemesis, dysphagia, unintentional weight loss, joint pain, past surgical history and a family history of inflammatory bowel disease). The term immigrant is used in this manuscript for patients, who recently migrated to Switzerland and therefore underwent a health screening.

### Statistical analysis

Means with standard deviations (SD) were calculated for each of the measurements of interest. To identify correlations between qualitative data Pearson’s chi-squared test and Fisher’s exact test were performed. Data entry and statistical analysis were performed using R, R commander and XLSTAT. A p-value of p < 0.05 was considered statistically significant.

### Ethical statement

The study was conducted in accordance to the ethical principles laid down in the Declaration of Helsinki and its later amendments. Furthermore, it was approved by the local ethical committee (Ethics committee of Northwest Switzerland, EKNZ, trial number 2018-02219).

## Results

A total of 1855 stool samples, belonging to 572 different children (56% female, 44% male) with an average age of 7.9 years, were tested within this 10-year period.

24 patients were found to have a positive result for parasites/helminths, which equates a prevalence of 4.2%. In all (24/24, 100%) patients with positive stool findings *Blastocystis* was detected, 6/24 (25%) of the patients had a co-infection with one (4/24, 16.6%) or several (2/24, 8.3%) other parasites, of which the most common one was *Endolimax nana* (3/24; 12.5%), see Table 1. None of the affected patients was immunosuppressed. 84/572 (14.7%) of the patients had their stool tested at the same time for viral infections, while in 20.8% (119/572) stool cultures were performed.

**Table 1:** Patient’s characteristics with positive and negative stool samples for parasitic/helminthic infections

Patients with positive stool samples			Patients with negative stool samples		
	Absolute	%		Absolute	%
*Sex*			*Sex*		
Male	15	63	Male	244	45
Female	9	38	Female	304	55
*Age*			*Age*		
Average age in years	9.1		Average in years	7.9	
0–2 years	0	0	0–2	70	12.8
2–6 years	7	29.2	2–6 years	141	25.7
6–9 years	5	20.8	6–9 years	116	21.2
9–12 years	4	16.7	9–12 years	106	19.3
12–15 years	3	12.5	12–15 years	83	15.1
15–18 years	5	20.8	15–18 years	30	5.5
18 +	0	0	18 +	2	0.4
*Origin*			*Origin*		
Swiss without history of travelling	11	45.8	Swiss without history of travelling	439	80.1
Swiss with history of travelling	3	12.5	Swiss with history of travelling	13	2.4
Immigrant	10	41.7	Immigrant	96	17.5
*Indication for stool testing*			*Indication for stool testing*		
Chronic abdominal pain	11	45.8	Abdominal pain	395	72.1
Immigrant health screening, no symptoms	9	37.5	Diarrhea	71	13
Diarrhea	4	16.7	Failure to thrive	17	3.1
*Results of stool findings*			*Flatulence*	10	1.8
Blastocystis	18	75	Vomiting	10	1.8
Blastocystis + Endolimax nana	2	8.2	Immigrant health screening, no symptoms	7	1.2
Blastocystis + E. nana + Chilomastix mesnili	1	4.2	Eosinophilia	6	1.1
Blastocystis + Enterobius vermicularis	1	4.2	Epigastric pain	5	0.9
Blastocystis + Pseudolimax bütschlii	1	4.2	Nausea	4	0.72
Blastocystis + G. lamblia + Entamoeba coli + Schistosoma mansoni	1	4.2	Other (perianal itchiness, encopresis, enuresis, iron deficiency, rash, fever, neutropenia, fever etc.)	23	4.2
*Treatment*			*Abdominal pain*	395	72.1
Treatment received (7 Metronidazole, 1 Mebendazole)	8	33.3	Diarrhea	71	13
*Final diagnosis*			*Symptoms persisted over 14 days*		
Functional pain/diarrhea	12	50	Yes	495	90.3
Incidental finding in the context of immigrant screening	9	37.4	No	49	8.9
			Not clear	4	0.8
Appendicitis	1	4.2	*Fever*		
Helminthiasis	1	4.2	Yes	20	3.6
Diarrhea in context of underlying inflammatory bowel disease	1	4.2	Abdominal pain	528	96.4
			*Final diagnosis*		
			Functional abdominal pain	396	72.3
			Viral/bacterial enteritis	36	6.6
			Toddler’s diarrhea	25	4.6
			Functional obstipation	17	3.1
			Other functional diseases (IBS, nausea, dyspepsia)	15	2.6
			Lactose-/Fructoseintolerance	11	2.1
			Diarrhea in context of new diagnosis of inflammatory bowel disease	9	1.6
			Postenteritis syndrome	5	0.9
			Others (allergies, eosinophil esophagitis, anorexia)	34	6.2

The correlation of an immigrant background and a positive result for Blastocystis was statistically significant (p-value = 0.02), but there was no significance calculated for the correlation of fever, duration of the symptoms, gender and the presence of parasites/helminths.

For those 24 patients with a positive stool result, on average 2.6 stool samples (a total of 64) per patient were analyzed for parasitical infections and a mean of 2.0 detected the parasites.

The majority (548/572; 95.8%) of all enrolled patients (55% female, 45% male; average age 7.9 years) had negative stool results for parasites/helminths and on average 3.2 stool samples per patient were analyzed.

Eighty percent of the patients with a negative screening for parasites/helminths were Swiss children without a history of travelling (see Table 1) and the main symptom was abdominal pain (72%), followed by diarrhea (13%), failure to thrive (3.1%), vomiting (1.8%) and flatulence (1.8%, see Table 1). 4 patients (4/548; 0.73%) with a negative stool test for parasites/helminths had a detection of bacterial (3/4 *Salmonella*, 1/4 *Clostridium difficile*) and 34 (34/548; 6.2%) of a viral (Adeno- and Rotavirus) infection.

Ninety percent of all negative tested patients complained about their main symptom for at least 14 days, 3% of them had a history of fever, whilst 4% of patients with a positive stool result suffered from fever.

## Discussion

We found a prevalence of 4.2% for parasitic/helminthic infections (all positive for Blastocystis) in our cohort of children/teenagers, which is in accordance of reports from other industrialized countries, such as Denmark with 5.6% [7] and the Netherlands [8] with around 20%. This number is clearly higher in developing countries [9]. In more than a third of the positive tested children, it was an incidental finding without any symptoms in the context of immigrant screening and in half of the cases the final diagnosis was functional pain, which has been associated with the presence of Blastocystis [10]. In the end, the finding of parasites only had a therapeutically consequence—namely a treatment with metronidazole or mebendazole—for a third of the positive tested patients. The debate about the clinical significance of Blastocystis is ongoing: So far 17 different genotypes (sub-types) of Blastocystis exist, which are found in different parts of the world. They are polymorphic in appearance -this might explain the different clinical presentation varying from incidental findings to severe abdominal complaints [11]. In our study sub-types of Blastocystis were not identified, as the polymerase chain reaction (PCR) method, which is needed for this process, was not used [12]. Once we know more about the pathogenicity, we might develop clear recommendations which Blastocystis sub-types we have to treat.

The second most commonly detected parasite was *Endolimax nana*, with a prevalence of 0.52%. Two case studies exist, which associate.

*Endolimax nana* with urticaria and polyarthritis, but there are no known cases of *Endolimax* crossing the intestinal barrier in humans, therefore it is speculated that it is apathogenic [13].

Another limitation of this study is that some, but not all of the patients had further stool tests performed for viral and/or bacterial infections.

Adding to a low positive detection rate of only 4.2%, might be the indication why stool was analyzed in the first place, which therefore became our main focus in analyzing the data. Looking at the cohort of pediatric patients with negative stool findings, it is striking that the main indication to perform the tests was abdominal pain (71%) and the most common final diagnosis (75%) was functional gastrointestinal disorders (FGIDs). Recurrent abdominal pain is a non-severe chronic medical condition which is defined as the presence of three or more episodes of abdominal pain over a period of at least 3 months that are sufficiently severe to affect daily activities [14] and is not due to an organic, structural or metabolic disease. FGIDs can be subdivided into esophageal disorders, functional nausea and vomiting disorders, functional abdominal pain and functional defecation disorders. The diagnosis and sub-classification of FGIDs is based on the Rome IV criteria [15], which were revised in 2016. Compared with the Rome III criteria, a relatively new concept is that clinicians no longer need to virtually exclude all organic causes of abdominal pain to make the diagnosis of FGIDs. The cornerstone of the diagnosis is a detailed anamnesis, laboratory test and radiological investigations are not mandatory for the diagnosis but should of course be considered when the physician recognizes some of the ‘red flag’ signs. The management of functional pain can remain a time-consuming and frustrating clinical challenge for physicians and gastroenterologists, especially when parents urge doctors for further investigation. However, it’s important to initiate not only reasonable diagnostics from a financial point of view, but also practicing a goal-oriented medicine, having in the back of our minds that in patients with a medical history without red flags and suggestive of a functional disease, we might only detect *Blastocystis*. This puts the clinician into the dilemma of making the decision ‘to treat or not to treat’ [16], as it is associated with functional disorders and the pathogenicity is unclear. Therefore, the European Rome IV criteria should be applied and only children with a clinical suspicion for a parasitic infection should be tested.

Twelve percent of all tested patients in our cohort were under the age of 2 years and we only found negative stool results for them. The main diagnosis after those tests was functional toddler’s diarrhea. The prevalence of this functional phenomenon was found to be 6% in the US [17]. When a child daily passes painlessly 4 or more large, unformed stools for at least 4 weeks, it has an adequate caloric intake without failure to thrive and is at onset of symptoms between 6 and 60 months of age, the diagnosis of functional toddler’s diarrhea can be made without further investigations according to the Rome IV Criteria for Functional Gastrointestinal Disorders in Infants and Toddlers [18]. Whilst there are lot of data proofing, that in developing countries parasites, such as *Cryptosporidium* are commonly causing episodes of diarrhea, especially in young children under the age of 5 years [19], the situation of enteric pathogens is different in Europe: Our findings are in line with a prospective study from Paris, where no parasites were detected in children under the age of 2 years [20].

In our cohort, the second most common indication in negative tested children for parasite infection was diarrhea. A disadvantage of this retrospective study is, that often the consistency and frequency of the stool is not specified, which is important to decide whether further diagnostic is indicated, as usually diarrhoeal illness has to be subdivided into three main categories, based on its clinical presentation: Acute watery, bloody or persistent diarrhea. According to the European Society for Pediatric Gastroenterology, Hepatology and Nutrition (ESPGHAN) and the European Society for Pediatric Infectious Diseases, the incidence of acute diarrhea (defined as a decrease in the consistency of stools and/or an increase in the frequency of evacuations, more than 3 in 24 h) ranges from 0.5 to 2 episodes per child per year in children under 3 years in Europe with Rotavirus being the most frequent agent [21]. The guidelines state that children presenting with acute gastroenteritis don’t require routine etiological investigation, but microbiological tests may be considered in children with immune deficiencies.

Bloody diarrhea is usually evidence of a bacterial infection (Salmonella, Shigella or enterohaemorrhagic *E. coli* pathotypes; [22–24]), but can also be caused by *Entamoeba histolytica.* Most infections are asymptomatic, but invasive intestinal disease may occur manifesting with several weeks of cramping, abdominal pain, bloody diarrhea and weight loss [25]. Persistent diarrhea lasts for at least 14 days can suggest a parasitic etiology, such as *Cryptosporidium species, Giardia intestinalis, Cyclospora cayetanensis, Dientamoeba fragilis, Cystoisospora belli* etc. [6]. A differential diagnosis of bloody and persistent diarrhea is inflammatory bowel disease and has to be ruled out [26].

3% of the negative tested children received the final diagnosis of functional constipation. The only situation, in which constipation can be caused by parasites is a partial/total blockage of the gut due to an excessive presence of them. In terms of size, *Ascaris lumbricoides*, is the largest roundworm that parasitize the human gastrointestinal tract and can cause constipation and intestinal obstruction in endemic regions, in patients with high worm loads. Although it is among the most common helminthic human infections with an estimated one billion people infected, it only exists in tropical and subtropical environments and should not be an indication to regularly test European children with signs of constipation [27].

Worldwide, areas with high rates of parasitic infection include India, Africa, and Central and South America [28], due to poor sanitation and socioeconomic conditions. Studies have reported that travelers to low- and middle-income countries (mainly areas in South America, Africa and South Asia) are between 9 and 151 times more likely to develop diarrhoeal illness [29, 30]. This is in line with our findings, where 80% of all patients with a negative stool result did not have a history of travelling abroad.

## Conclusion

Although data regarding helminth and parasitic infections amongst European children is still sparse, based on our data stool examinations are performed more often than needed relying frequently on a loose indication. Immune competent children suffering from acute diarrhea -especially under the age of 2 years-, constipation or from abdominal pain without diarrhea and without history of travelling, should not be tested. Even though stool testing is not invasive it should be only applied in patients with a risk factor. In children with a suggestive anamnesis of functional disorders (including functional toddler’s diarrhea), clinicians should refer to the Rome IV Criteria and abstain from pro forma stool examinations. The probability of a negative result in those patients is high and one might only detect *Blastocystis* or *Endolimax nana,* whose clinical consequence is debatable. Immune compromised children, immigrants or pediatric patients with bloody or protracted diarrhea should have 2 different stool specimens examined.

## Data Availability

The dataset used/analyzed during the current study is available from the corresponding author on reasonable request.

## Abbreviations

ESPGHAN: European Society for Pediatric Gastroenterology, Hepatology and Nutrition
FGID’s: Functional gastrointestinal disorders
